# Inclusive physical activity games at school: The role of teachers’ attitude toward inclusion

**DOI:** 10.3389/fpsyg.2023.1158082

**Published:** 2023-03-29

**Authors:** Ambra Gentile, Valerio Giustino, Olga Rodriguez-Ferrán, Alessandra La Marca, Giuseppa Compagno, Antonino Bianco, Giuseppe Battaglia, Marianna Alesi

**Affiliations:** ^1^Department of Psychology, Educational Sciences, and Human Movement, University of Palermo, Palermo, Italy; ^2^Facultad de Ciencias del Deporte, Campus de Excelencia Internacional Mare Nostrum, Universidad de Murcia, Murcia, Spain

**Keywords:** inclusive education, social desirability, attitude toward inclusion, familiarity, teachers’ self-efficacy

## Abstract

**Introduction:**

Inclusive physical activity games at school can be useful for teachers dealing with students with disabilities. The use of inclusive strategies and games can be directly linked to teachers’ self-efficacy and familiarity with the inclusive strategies, while it could be indirectly influenced by their attitude toward inclusion and, in a smaller part, by social desirability in their response. Moreover, teachers’ responses could be different among the different school grades. Therefore, the aim of the current study is to investigate the role of attitude toward inclusion, social desirability, self-efficacy, and familiarity in the use of physical activity games at school in teachers from all school grades.

**Methods:**

A sample of 1,583 schoolteachers was asked to fill out a questionnaire about their perceptions of governmental measures, self-efficacy, familiarity with inclusive strategies through physical activity, and two standardized questionnaires assessing attitudes toward inclusion and social desirability.

**Results:**

Teachers from primary school reported lower scores in attitude toward inclusion total score and dimensions, namely impact on teacher, impact on the environment, impact on the other children, and impact on the student with disability. Moreover, the path analysis model showed that the attitude toward inclusion indirectly influenced the use of inclusive strategy and had a small direct effect on familiarity with inclusive strategies and self-efficacy. Social desirability slightly influenced both familiarity and self-efficacy but not the use of inclusive strategies. Familiarity and self-efficacy had a direct effect on the use of inclusive physical activity games.

**Discussion:**

The results of the current study suggest that being familiar with and having a high self-efficacy in implementing inclusive strategies are well related to the use of inclusive strategies at school. In addition, more attention should be given to kindergarten and primary school teachers, who reported lower scores in the attitude toward inclusion and higher scores in social desirability.

## 1. Introduction

Inclusive education is a priority in modern societies, increasingly moving toward social justice and equity (Nes et al., 2018). The definition of inclusive education as essential for children’s wellbeing was promoted by the Salamanca Statement (UNESCO, 1994). Concerning its definition, the concept of inclusive education is sometimes broad and general. Some authors defined it as integrating students with special needs into regular classes, while others referred to the school system as a welcoming place for all the students (Ainscow et al., 2006; Nes et al., 2018).

One of the tools suitable for implementing inclusive strategies is physical activity (PA). It is well-known that physical activity brings physical, cognitive, and psychosocial benefits to children and adolescents with both typical and atypical development (Janssen and LeBlanc, 2010; Bidzan-Bluma and Lipowska, 2018; Rodriguez-Ayllon et al., 2019). Regarding disability, PA was demonstrated to positively affect general health, including physical and mental health (Donnelly et al., 2016). Concerning physical health, improvements were found in physical fitness, bone metabolism, cardiovascular and respiratory muscle functions, and weight control (Marsigliante et al., 2023; Salvadego et al., 2023). Regarding benefits on mental health, the improvements were retrieved in terms of an increase in self-esteem, self-efficacy, and positive self-perception and terms of social domain such as functional independence and social inclusion (Bull et al., 2020; Virgara et al., 2021). In particular, the social benefits of physical activity could enhance residual potentialities in severe grade disabilities by improving motor autonomy and perceptive awareness, in moderate grade disabilities by helping the mastery of basic motor skills, and in mild grade disabilities by facilitating the mastery of complex motor skills to practice sport activities (Escalante et al., 2014; Chaput et al., 2020). Moreover, the production of neurotrophins, synaptogenesis, and angiogenesis, particularly in the prefrontal cortical area, cognitive abilities, such as speed of processing, working memory, planning, and control strategies, are reinforced (Jones et al., 2010).

The psychological literature has identified the role of the attitude toward inclusion as a prerequisite for implementing inclusive strategies and games at school (Avramidis and Norwich, 2002). As pointed out by Lüke and Grosche (2018), most of the studies using blatant measures to detect the attitude toward inclusion barely considers the influence of social desirability. This bias may occur especially in countries with high sensitivity toward minorities and people with disability, where the social norm on inclusion is quite clear. In this way, the risk is that the answers to blatant questionnaires upon inclusion could be much more positive due to the need to look good in front of other people. Research has also focused on the comparison of the attitude toward inclusion across the school grades, with teachers from primary schools having a more negative attitude than secondary school teachers (Barnes and Gaines, 2015; Gaines and Barnes, 2017).

Concerning the use of inclusive strategies at school, teachers’ self-efficacy was revealed to be a strong predictor of inclusive practice implementation in school classes (Acedo et al., 2009). Furthermore, a study by Kiel et al. (2020) reported that teachers with higher levels of self-efficacy also referred to implement inclusive strategies to a greater extent. Regarding its relationship with attitude toward inclusion, a study by Werner et al. (2021) found that teachers’ self-efficacy was related to a higher attitude toward inclusion and was influenced by the knowledge of school policies and the support given by the school concerning the inclusion matter.

Moreover, teachers’ familiarity with skills and teaching strategies directly affects the implementation of these strategies (Schiepe-Tiska et al., 2021). In other words, being familiar with instructional strategy can lead to a better ability to design activities for students with disability (Kirch et al., 2005). Therefore, familiarity with inclusive strategy should be linked to the real implementation of these strategies.

To our knowledge, no studies explored the influence of teachers’ attitudes toward inclusion, social desirability, self-efficacy, and inclusive strategies familiarity on the implementation of physical activity games in teachers from all school grades. Therefore, the current study aims to fill this gap and hypothesizes a direct effect of teachers’ self-efficacy and familiarity with inclusive physical activity games on the use of inclusive games and an indirect effect of attitude toward inclusion and social desirability in teachers’ responses.

## 2. Materials and methods

### 2.1. Participants

Participants were recruited with convenience sampling by sharing the questionnaire through social networks with the target group and asking respondents to spread the questionnaire. A sample of 1,583 teachers belonging to the different school orders (i.e., kindergarten, primary school, middle school, and high school) completed the survey. The majority of teachers belonged to primary school (*n* = 559, 35.3%), secondary school (*n* = 486, 30.7%), and high school (*n* = 409, 25.8%), while a small part of the sample was made up of kindergarten teachers (*n* = 128, 8.1%). The study was conducted respecting the principles of the Declaration of Helsinki, and the Bioethics Committee of the University of Palermo approved the study (protocol nr. 63/2021).

### 2.2. Measures

For this cross-sectional study, an online survey through the Google Forms web survey platform (Google LLC, Mountain View, CA, USA) was employed. The survey was conducted from October to December 2021. At the beginning of the survey, a brief description of the study was provided. The survey consisted of 54 items related to inclusive physical activity games at school, assessing the perceptions of teachers about school and governmental policies on inclusion, the ability to implement and the familiarity with inclusive physical activity games and inclusive strategies in the classroom, a standardized measure concerning the attitude toward inclusion at school, and a standardized measure concerning social desirability.

#### 2.2.1. Inclusive physical activity at school

The first section comprised: (a) data concerning the school context (e.g., “The promotion of inclusion in my school consists of school management measures”); (b) perceived policies and norms on inclusion adopted by government (“The promotion of inclusion in my school consists of governmental measures”); (c) perceived self-efficacy in managing inclusive physical activity games (“I am able to use physical activity games/inclusive strategies in my classroom,” “I have familiarity with physical activity games/inclusive strategies in my classroom”); (d) use of inclusive physical activity games in the classroom (“I use inclusive physical activity games/inclusive strategies in my classroom”). The items were assessed on a 7-point Likert scale from 1 (“completely disagree”) to 7 (“completely agree”).

#### 2.2.2. Attitude toward inclusion

The attitude toward inclusion was assessed through an Italian adaptation of the Impact of Inclusion Questionnaire (Hastings and Oakford, 2003), which is a 24-items self-report questionnaire on a 7-point Likert Scale (from 1 = Very Strongly Disagree to 7 = Very Strongly Agree). The questionnaire examines the impact of inclusion on teacher (“having children with disability in my classroom physically wears me out”), on the environment (“having children with disability in my classroom interrupts the classroom routine”), on other children (“having children with disability in my classroom increases other children’s problematic behavior”), and on children with disability (“having children with disability in my classroom does not encourage their difficult behavior”). Higher scores indicated a more positive attitude toward inclusion. The internal reliability of the scale was good (α = 0.82).

#### 2.2.3. Social desirability

Social desirability was assessed through an Italian adaptation of the Marlowe–Crowne Social Desirability Scale–Short form C (MC-C) (Reynolds, 1982). The scale consists of 13 items with dichotomic answers Yes/No and assesses the attitude toward general situations that may be sensitive to social desirability (“I am always courteous, even to people who are disagreeable”). The internal reliability of the scale was not very high (α = 0.54), but as reported by Ray (1984), it may happen in short forms of social desirability scales.

### 2.3. Data analysis

Data were analyzed through Statistical Package for the Social Sciences (SPSS; IBM, version 24). Descriptive statistics were performed on the sample, distinguishing the school grades’ scores. Next, an ANOVA model was performed to detect differences in school grades concerning inclusive education, the impact of inclusion, and social desirability. Differences across groups were detected through the Bonferroni *post-hoc* test. We determined how social desirability affected the attitude toward inclusion scores through the ANCOVA model. Then, intercorrelations among attitude toward inclusion perceived governmental and school measure, perception of competence concerning inclusion, and use of inclusive strategies were performed using Pearson’s r. Finally, a path analysis model was performed through MPlus (Version 7) (Muthén and Muthén, 2017).

## 3. Results

### 3.1. Descriptive statistics

Descriptive statistics are presented in Tables 1, 2. The scores for the governmental and school management measures, perception of competence in implementing inclusive strategies, familiarity with inclusive strategies, and use of inclusive strategies are presented in Table 1.

**TABLE 1:** Descriptive statistics of governmental measures, school measures, familiarity, self-efficacy, and use of inclusive games.

School grades	Kindergarten (*n* = 128)		Primary school (*n* = 559)		Middle school (*n* = 486)		High school (*n* = 409)		
	*M*	SD	*M*	SD	*M*	SD	*M*	SD	*F*
Governmental measures in my school	4.92	1.13	5.01	1.12	4.96	1.24	4.88	1.19	0.92
Measures from the school management	5.27	0.93	5.34	1.04	5.38	1.12	5.22	1.11	1.93
I am able to use physical activity games/inclusive strategies in my classroom	5.22	0.97	5.41	1.06	5.13	1.08	5.21	1.00	6.80***
I have familiarity with physical activity games/inclusive strategies in my classroom	5.33	0.87	5.41	1.01	5.10	1.09	5.13	1.04	9.97***
I use inclusive physical activity games/inclusive strategies in my classroom	5.54	0.98	5.58	0.99	5.35	1.14	5.33	1.03	5.55**

**p < 0.01, ***p < 0.001.

**TABLE 2:** Descriptive statistics of attitude toward inclusion and social desirability.

School grades	Kindergarten (*n* = 128)		Primary school (*n* = 559)		Middle school (*n* = 486)		High school (*n* = 409)		
	*M*	SD	*M*	SD	*M*	SD	*M*	SD	*F*
Impact of inclusion (total score)	122.38	14.88	124.80	13.95	128.06	13.20	126.56	13.62	11.70***
Impact on teacher	32.71	5.62	33.28	5.79	34.29	5.53	34.04	5.53	4.60***
Impact on the environment	34.02	4.81	34.70	4.69	35.59	4.41	35.47	4.20	6.74***
Impact on the other children	28.41	4.53	29.10	4.30	29.10	4.12	29.65	4.16	5.01***
Impact on children with disability	27.45	3.15	27.64	3.15	27.64	2.75	28.71	3.00	16.62***
Social desirability	23.78	1.65	24.20	1.55	23.85	1.89	23.72	1.83	7.21***

***p < 0.001.

No differences were detected concerning governmental and school measures across the school grades. Significant differences among groups were detected in the perception of competence (*F*_3,1577_ = 6.80, *p* < 0.001), familiarity (*F*_3,1577_ = 9.97, *p* < 0.001), and use of inclusive strategies (*F*_3,1577_ = 5.55, *p* < 0.01). From the *post-hoc* comparison, the perception of competence was higher in primary school teachers than in middle (MD = 0.28, *p* < 0.001) and high school teachers (MD = 0.20, *p* = 0.02). Similarly, familiarity was higher in primary school teachers than middle (MD = 0.31, *p* < 0.001) and high school (MD = 0.28, *p* < 0.001). The same pattern emerged concerning the use of inclusive strategies, where primary school teachers reported higher scores than middle (MD = 0.21, *p* < 0.01) and high school teachers (MD = 0.23, *p* < 0.01).

### 3.2. Attitude toward inclusion

The dimensions of the Impact of Inclusion Questionnaire and the results of the Marlowe–Crowne Social Desirability Scale are presented in Table 2.

Significant differences across the groups emerged in the total score (*F*_3,1577_ = 11.70, *p* < 0.001) and in all the dimensions of the Impact of Inclusion Questionnaire (Teacher: *F*_3,1577_ = 4.60, *p* < 0.01; Environment: *F*_3_,1577 = 6.74, *p* < 0.001; Other: *F*_3,1577_ = 5.02, *p* < 0.01; Children with disability: *F*_3,1577_ = 16.62, *p* < 0.001), as well as in social desirability scores (*F*_3,1577_ = 7.21, *p* < 0.001). From the *post-hoc* comparisons, the impact of inclusion on teacher was higher in middle school teachers than in kindergarten (MD = 1.58, *p* = 0.03) and primary school teachers (MD = 1.01, *p* = 0.02). Concerning the impact of inclusion on the environment, the scores were higher for teachers at middle school than teachers from kindergarten (MD = 1.58, *p* < 0.01) and primary school (MD = 0.89, *p* < 0.01), and for teachers from high school than those from kindergarten (MD = 1.45, *p* < 0.01). Regarding the impact on the other children, the scores were higher in middle school (MD = 1.36, *p* = 0.03) and high school teachers (MD = 1.24, *p* < 0.01) than in kindergarten teachers. Concerning children with disability, the scores were higher in middle school and high school than in kindergarten teachers (middle school: MD = 1.19, *p* < 0.001; high school: MD = 1.26, *p* < 0.01), and than primary school (middle school: MD = 1.00, *p* < 0.001; high school: MD = 1.07, *p* < 0.01).

### 3.3. The influence of social desirability on the attitude toward inclusion

Concerning social desirability, the scores were higher in primary school than in middle school (MD = 0.35, *p* < 0.01) and high school teachers (MD = 0.49, *p* < 0.001).

From the ANCOVA models, the total score and three out of the four dimensions of the Impact of Inclusion were sensitive to social desirability. In particular, the impact on teacher (*F* = 247.13, *p* < 0.001; *R*^2^ = 0.14) was the most sensitive, followed by the total score (*F* = 216.18, *p* < 0.001; *R*^2^ = 0.14), then the impact on the environment (*F* = 159.91, *p* < 0.001; *R*^2^ = 0.08), and finally on the other children (*F* = 126.76, *p* < 0.001; *R*^2^ = 0.08). Social desirability did not affect the impact of inclusion on children with disability (*F* = 1.48, ns; *R*^2^ = 0.03).

### 3.4. Path analysis model

The correlation matrix of Table 3 shows the intercorrelations among the investigated variables. All the variables showed a mild correlation.

**TABLE 3:** Intercorrelations among the considered variables.

	1	2	3	4	5
Attitude (1)	−				
Social desirability (2)	0.34***	−			
Self-efficacy (3)	0.21***	0.21***	−		
Familiarity (4)	0.18***	0.21***	0.73***	−	
Use of inclusive games/strategies (5)	0.23***	0.18***	0.64***	0.66***	−

***p < 0.001.

A path analysis model was performed with attitude toward inclusion and social desirability influencing the perception of competence in inclusive strategies and familiarity with inclusive strategies, that in turn influence the use of inclusive strategies during the school classes.

**FIGURE 1:**
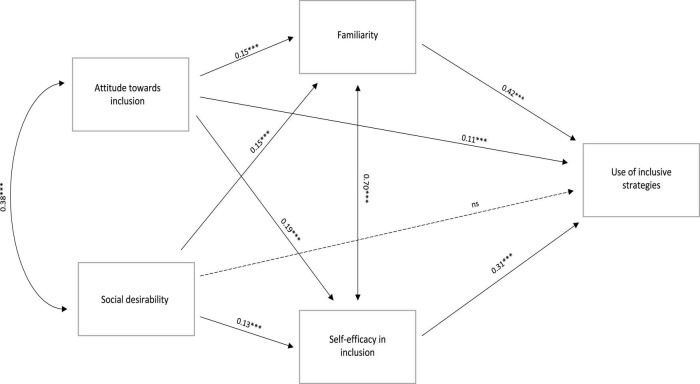
Path analysis model. ***p < 0.001. ns, non significant.

The model (Figure 1) showed an excellent fit (χ^2^ = 69.143, df = 14; CFI = 0.98, RMSEA = 0.05, 95% CI: 0.04−0.06, SRMR = 0.03). Attitude toward inclusion (β = 0.15) and social desirability (β = 0.13) slightly influenced the perception of competence and the familiarity with inclusive strategies (perception of competence: β = 0.19, familiarity: β = 0.15). The perception of competence (β = 0.33) and familiarity (β = 0.42), in turn, influenced the use of inclusive strategies during school classes. Attitude toward inclusion slightly influenced the use of inclusive strategies (β = 0.11) and correlated with social desirability scores (β = 0.38), while familiarity with inclusive strategies strongly correlated with the perception of competence (β = 0.70). Social desirability did not directly influence the use of inclusive strategies. In other words, having a positive attitude toward inclusion and having the tendency to be socially desirable, together with the perception of being competent and familiar with inclusive strategies, influence the probability of using the inclusive strategy at school.

## 4. Discussion

The current study aimed to explore the role of the attitude toward inclusion, social desirability, teachers’ familiarity with inclusive games, and self-efficacy in implementing physical activity games in ordinary classes. Specifically, we first assessed the differences between teachers across the school grades concerning the perception of school and governmental support about inclusion, self-efficacy and familiarity with inclusive strategies, attitude toward inclusion dimensions, and social desirability. We then hypothesized a direct effect of teachers’ self-efficacy and familiarity with inclusive physical activity games on the use of inclusive games and an indirect effect of attitude toward inclusion and social desirability in teachers’ responses.

Concerning the first research question, no differences were found concerning perceptions of governmental and school measures across the school grades, while the perception of competence was higher in primary school teachers than in middle and high school teachers. Similarly, familiarity was higher in primary school teachers than in middle and high school teachers, as well as the use of inclusive strategies. Our results are in line with the ones from a study by Triviño-Amigo et al. (2022), reporting that primary school teachers felt more prepared to implement inclusive strategies and reported to have received good training for inclusive systems than secondary school teachers.

In our study, concerning the attitude toward inclusion, a significantly lower score was reported by kindergarten and primary school teachers compared to middle and high school teachers. Similar results were reported in a study by Gaines and Barnes (2017) that found less positive scores toward inclusion in primary school teachers than in secondary school. Also, secondary and high school teachers tend to report more positive attitudes toward inclusion, whereas high school teachers displayed the most positive attitude toward inclusion (Galović et al., 2014; Schmidt and Vrhovnik, 2015). Taken together, these results indicate that the attitude toward inclusion is more positive in the higher school grades, probably because of the increased number of students with special needs in primary school classes compared to the other grades (Gaines and Barnes, 2017). Indeed, teachers with fewer special needs students tend to report a more positive attitude toward inclusion (Galović et al., 2014). In addition, primary school represents a challenging stage for children’s development because, during this period, children are expected to adapt for the first time to teacher and classroom demands, especially for children with special needs (McIntyre et al., 2006; Symeonidou and Phtiaka, 2009). In this way, teachers might experience higher levels of stress and be less positive toward inclusion compared to teachers from the other school grades.

Moreover, primary school teachers reported higher scores on social desirability than secondary and high school teachers. As reported by Tempel (2022), social desirability should be considered when assessing sensitive attitudes through self-reported measures, especially in countries with high sensitivity toward minorities and disabilities (Lüke and Grosche, 2018). In our study, social desirability is likely to affect most primary teachers’ responses, which already have lower scores concerning the attitude toward inclusion.

A path analysis model was performed to understand the extent to which attitudes toward inclusion and social desirability influence teachers’ familiarity and self-efficacy and, in turn, the use of inclusive strategies. Specifically, we found a moderate correlation between attitude toward inclusion and social desirability, which, in turn, were slightly associated with teachers’ familiarity with inclusive strategies and self-efficacy. Both familiarity and self-efficacy displayed a moderate association with the use of inclusive strategies and were strongly associated with each other. Moreover, attitude toward inclusion slightly influenced the use of inclusive practices, while social desirability did not influence the use of inclusive strategies.

Our model’s results indicate that the relationship between attitude toward inclusion and social desirability are not strongly related or are not related at all with the use of inclusive practices. Similarly, Sharma et al. (2021) found that the attitude toward inclusion has a weak association with the use of inclusive strategies.

Moreover, in our model, being familiar and confident with the use of inclusive strategy positively influences the use of inclusive strategies. Indeed, self-efficacy has been identified as one of the best predictors of the use of inclusive strategies (Sharma et al., 2021), and our data are in line with this finding.

Our study has the advantage to explore the relationship among the overmentioned variables in a big sample of teachers. However, it comes out with some limitations: first, the study is correlational, so it is not possible to draw causal relationships. Furthermore, we did not assess the behavioral intention, which should be the strongest predictor of behavior according to the Theory of Planned Behavior (Ajzen, 1991). Finally, we did not assess the influence of teaching experience, that could be a variable affecting the attitude toward inclusion, as teachers without experience might face more stress compared to teachers who have consolidated experience at school.

The results of the current study suggest that being familiar with and having a high self-efficacy in implementing inclusive strategies are well related to the use of inclusive strategies at school. Therefore, University curricula should focus more on training the future teachers about the inclusion matter, especially for what concerns the management of different types of disability in the same school class.

Several studies reported a lack of knowledge and expertise in the class management in case of disability, using tools such as physical activity or technology (Aihie and Uwaoluetan, 2022; Nanayakkara, 2022; Tarantino et al., 2022). Physical activity games could be a good tool for children’s health, considering that children with disability barely practice physical activity and are at more risk of obesity (Alesi et al., 2022; Alghamdi and Alsaigh, 2023). Therefore, teachers should be involved in formal and informal training concerning the strategies for adapting physical education games to different disabilities.

Furthermore, a deeper analysis concerning the way teachers live the inclusion matter should be conducted. Even though the attitude toward inclusion is weakly associated with using inclusive strategies, other variables might be related to a not-so-positive attitude toward inclusion. Teaching is a stressful job that became even more stressful during the COVID-19 pandemic. According to a study of Salinas-Falquez et al. (2022) conducted during the COVID-19, attitude toward educational inclusion was negatively associated with burnout. Similarly, in another study, teachers scoring low levels of attitude toward inclusion also have high scores of stress (Avramidis and Norwich, 2002). In addition, another study pointed out that the stress experienced within the classroom was a significant predictor of a negative attitude toward inclusion (Galaterou and Antoniou, 2017).

Conversely, teachers receiving a training about inclusion show a more positive attitude toward inclusion, a better cognitive and emotional appraisal, and higher levels of self-efficacy (Dignath et al., 2022).

Therefore, future research should better investigate how to improve working conditions for teachers who experience the burden of inclusive education at all levels. From the policy side, an improvement of in-service teachers’ training conditions and contents is required, to guarantee a positive attitude at work and to avoid negative consequences for children’s and teachers’ health.

## Data availability statement

The datasets presented in this article are not readily available because data are available under formal request to the Bioethics Committee of the University of Palermo. Requests to access the datasets should be directed to segreteria.comitato.bioetica@unipa.it.

## Ethics statement

The current study has been approved by the Bioethics Committee of the University of Palermo (protocol nr. 63/2021). The patients/participants provided their written informed consent to participate in this study.

## Author contributions

AG, VG, and MA: conceptualization and formal analysis. AG, AB, and MA: methodology. AG, VG, and GB: software. GC and AL: investigation. AG, VG, MA, GC, and AL: writing—original manuscript. AB and GB: writing—review and editing. All authors approved the final version of the manuscript.
